# A new cost-effective method to mitigate ammonia loss from intensive cattle feedlots: application of lignite

**DOI:** 10.1038/srep16689

**Published:** 2015-11-20

**Authors:** Deli Chen, Jianlei Sun, Mei Bai, Kithsiri B. Dassanayake, Owen T. Denmead, Julian Hill

**Affiliations:** 1 Faculty of Veterinary and Agricultural Sciences, The University of Melbourne, Victoria 3010, Australia; 2 Ternes Agricultural Consulting Pty Ltd, Upwey, Victoria 3158, Australia

## Abstract

In open beef feedlot systems, more than 50% of dietary nitrogen (N) is lost as ammonia (NH_3_). Here we report an effective and economically-viable method to mitigate NH_3_ emissions by the application of lignite. We constructed two cattle pens (20 × 20 m) to determine the effectiveness of lignite in reducing NH_3_ emissions. Twenty-four steers were fed identical commercial rations in each pen. The treatment pen surface was dressed with 4.5 kg m^−2^ lignite dry mass while no lignite was applied in the control pen. We measured volatilised NH_3_ concentrations using Ecotech EC9842 NH_3_ analysers in conjunction with a mass balance method to calculate NH_3_ fluxes. Application of lignite decreased NH_3_ loss from the pen by approximately 66%. The cumulative NH_3_ losses were 6.26 and 2.13 kg N head^−1^ in the control and lignite treatment, respectively. In addition to the environmental benefits of reduced NH_3_ losses, the value of retained N nutrient in the lignite treated manure is more than $37 AUD head^−1^ yr^−1^, based on the current fertiliser cost and estimated cost of lignite application. We show that lignite application is a cost-effective method to reduce NH_3_ loss from cattle feedlots.

Ammonia (NH_3_), a form of reactive nitrogen (N), poses negative effects on ecosystems and biodiversity through its deposition, on human health through secondary particulate matter formation, and on emissions of the greenhouse gas nitrous oxide^1^,^2^. Globally, livestock industries account for as much as 40% of total NH_3_ emissions^3^. Cattle feedlots are large hotspots of NH_3_ and about 53–65% of the N consumed in feedlot rations is lost as NH_3_^4^,^5^. It is suggested that the feedlot pen is the major source of NH_3_ emissions from cattle feeding operations as faeces and urine are deposited directly to the surface and urinary urea (50 to 90% N in urine^6^) is rapidly hydrolysed into NH_3_ and then lost to the atmosphere via volatilization.

Strategies to mitigate NH_3_ emissions from feedlots have been suggested, which include changing diet formulation^7^,^8^, and using additives or management to alter soil and storage conditions of manure to suppress urea hydrolysis^9^,^10^,^11^. However, none of these approaches have been adopted widely by the industry, because of cost and/or difficulties in on-farm implementation of those practices in commercial environments.

Lignite (brown coal) is a low rank, low ash, high moisture content coal^12^. There are large reserves of lignite in the Latrobe Valley of Victoria, Australia. This lignite is acidic in nature, has a high humic acid content, high cation exchange capacity and contains up to 20% of labile carbon, all of which may suppress NH_3_ volatilization from manure. It has been reported that NH_3_ emissions can be significantly reduced with acidifying additives^13^. For instance, 60–68% NH_3_ reduction from cattle manure by brown/black humate application was reported by Shi *et al.*^14^. The use of lignite in abating NH_3_ emissions from open feedlot pens is conceptually promising, but has not been previously reported. We conducted an experiment at Dookie (36.39°S, 145.71°E), Victoria, Australia, to quantify the abatement potential of lignite application on NH_3_ emissions from feedlots. We used two cattle pens each holding 24 black Angus steers and measured NH_3_ concentrations continuously for 40 days using Ecotech EC9842 NH_3_ analysers in conjunction with a mass balance method to calculate NH_3_ fluxes.

## Results and Discussion

**Figure 1:**
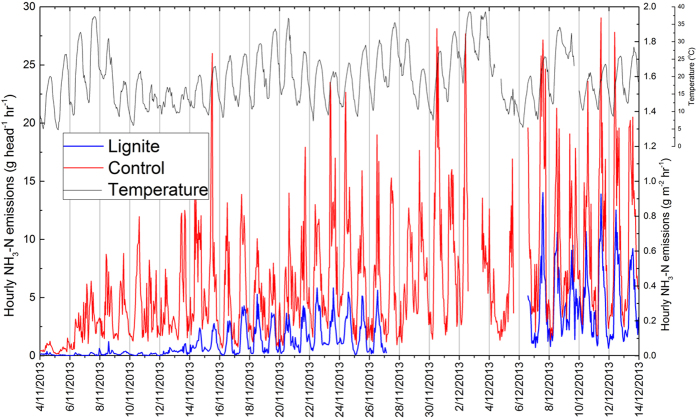
Hourly NH_3_-N emissions and air temperature from 4^th^ November to 13^th^ December. Cattle moved in pens at 9–11 am on 4^th^ November and moved out at 1–3 pm on 13^th^ December.

A strong diurnal variation in NH_3_ emissions from both pens was observed, with the lowest emissions occurring at dawn and the highest occurring at around mid-day (Fig. 1). This pattern in emissions corresponds to the daily temperature variation and has been reported in other studies^2^,^15^. Hourly emission rates of NH_3_ varied from 0.01 to 14.0 g N head^−1^ hr^−1^ for lignite treatment, and from 0.14 to 29.0 g N head^−1^ hr^−1^ for control treatment. Ammonia emissions from the control pen increased significantly after cattle were introduced (9–11 am on 4^th^ November) (Fig. 1), reflecting rapid hydrolysis of urinary-urea^16^,^17^. Ammonia volatilization was almost completely suppressed by lignite during the first 10 days compared to the control. After that, the suppression started to decline, but the NH_3_ emission rates in the lignite treated pen were still about 50% less than that in control pen (Fig. 1) at the end of (40 days) experiment.

**Figure 2:**
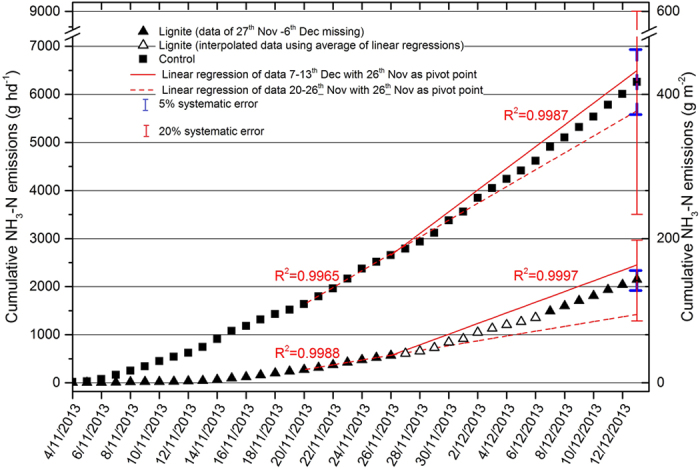
Cumulative NH_3_-N emissions from 4^th^ November to 13^th^ December.

The average daily NH_3_ emission rates were 53.2 ± 6.4 and 156.4 ± 10.7 g N head^−1^ d^−1^ for lignite and control pens, respectively (Table 1). The NH_3_ emission rate from the control pen was comparable to those observed in other feedlot studies (100−200 g N head^−1^ d^−1^)^15^,^18^. Nitrogen excretion from the cattle was estimated to be approximately 350 g head^−1^ d^−1^ (using NRC^19^ estimates). Nitrogen loss through NH_3_ volatilization from pen surface accounted for approximately 15 and 45% of N in cattle excretion, for lignite and control pens, respectively. The application of lignite reduced NH_3_ emission by 103.2 g N head^−1^ d^−1^ or 66.0% compared to the control. The cumulative NH_3_ emissions were 2.13 ± 0.11 and 6.26 ± 0.31 kg N head^−1^, for lignite and control pens, respectively (Table 1 and Fig. 2). When collected from pens after 40 days, manure treated with lignite had a higher N content (2.4%) than that of the control pen (1.7%). The amount of N retained in manure was 9.9 and 5.3 kg head^−1^ for lignite and control pen, respectively.

Our results show that application of lignite is more effective, practical and longer lasting than applying the urease inhibitor NBPT (47–49%^17^ or 64–66%^14^ reduction of ammonia loss, last less than a week^17^, and not tested for continuous excretion-N input at feedlots), humate^14^ (60–68% reduction of ammonia loss, high application rate and not cost effective) or acidifying additives^11^ (normally require complex application systems). Lignite abates NH_3_ emissions through its strong acidity^13^,^20^ (pH 3.69), strong adsorption capacity of ammonium^20^ (cation exchange capacity 96.8 cmol(+) kg^−1^) as well as biological immobilisation due to the high content of labile carbon^21^,^22^ (20.1%). The humic acid content of the lignite may also indirectly inhibit urea hydrolysis^23^. However, these effects will decline when the acidity is neutralised and the cation exchange capacity reduced through the accumulation of manure in the feedlot. After routine manure removal from pens, lignite needs to be reapplied to optimise the reduction of NH_3_ emissions.

It has been widely reported that the application of feedlot manure to crop land can increase crop yield, maintain soil organic matter content, and improve soil physical condition^24^,^25^. Feedlot manure with higher N content can practically reduce the total application amount, resulting in less environmental risks related to other nutrients in manure, such as leaching of phosphorus^26^. When extrapolating to an annual basis, the addition of lignite decreased NH_3_ volatilization by approximately 38 kg N head^−1^ yr^−1^. Given the market price for urea fertiliser (46% N) of $600 AUD tonne^−1^, the N nutrient retained in the manure by lignite is equivalent to approximately $49 AUD head^−1^ yr^−1^. We estimate the cost of lignite application at a commercial feedlot, including cost of purchase, transportation of 500 km from source, and feedlot surface dressing of 4.5 kg dry mass applied every 40 days, to be $11.7 AUD head^−1^ yr^−1^.

The emitted NH3 from intensive sources may have substantial local impacts on the surrounding ecosystems^27^,^28^. A study of NH3 deposition near a feedlot in Canada revealed that a large portion (19%) of emitted NH3 was deposited within 1.7 km of the source^29^. Therefore, reducing emissions from the local hot spots such as feedlots will also achieve local environmental benefits. In summary, the addition of lignite is a cost-effective method for mitigating NH3 emissions, reducing environmental impacts and improving N use efficiency of these intensive animal production systems. These findings have major economic and environmental implications for effective N management in agriculture, especially in feedlots.

## Methods

The experimental site was topographically flat and underlain by a clay soil. The prevailing winds during the experiment period were SSW, with the minimum daily temperature 6 °C and the maximum 39 °C (Fig. 1). Two cattle pens (20 × 20 m, 180 m apart) were constructed to mimic the environment of cattle feedlots. Prior to introducing animals, lignite, at a rate of 4.5 kg m^−2^, was spread uniformly within the treatment pen. The lignite, Yallourn Brown Coal, had a pH of 3.69, a cation exchange capacity of 96.75 cmol(+) kg^−1^, a labile carbon content of 20.13% and a water content of 65%. No lignite was applied in the control pen. Twenty-four Angus steers (*Bos taurus*; 12 months of age, with initial average live weight of 486 ± 33 kg) were put into each pen. Ammonia flux measurements were conducted from 4^th^ November (cattle moved in around 9–11 am) to 13^th^ December 2013 (cattle moved out around 1–3 pm) for 40 days. During this period the cattle were fed twice a day with a diet of 50% grain and 50% hay (17% crude protein, 27.2 g N kg^−1^ dry matter). Live weight of cattle and the weight of accumulated manure were recorded at the end of the measurement period. These data were used to estimate N excretion of urine and faeces using NRC^19^. All experiments were approved by the University of Melbourne Animal Ethics Committee under licence 1312794.1 and conducted in accordance with guidelines and regulations of this committee.

An NH_3_ chemiluminescence analyser (EC9842, Ecotech Pty Ltd, Australia) was used to measure NH_3_ concentrations at each pen. The analysers were housed in air-conditioned trailers and placed approximately 30 m away from the pens. Analysers were calibrated against an NH_3_ target tank every two weeks. Air was transferred to the NH_3_ analysers through ¼ inch OD Teflon tubing from a sampling mast in the centre of each pen. There were 5 sampling inlets at heights of 0.25, 1, 2, 3 and 4 m. Sampling lines were constantly pumped and samples were delivered to the analysers via an automated manifold with a sequenced switching program. Every inlet was sampled for 6 minutes, resulting in a half-hour cycle of the five inlets. A custom-made hot water sleeve system was used to maintain temperatures of sampling lines at 45 °C to prevent NH_3_ condensation or build-up in the sampling lines. A two-dimensional sonic anemometer (WindSonic, Gill Instruments Ltd, UK) was mounted at each sampling height to record horizontal wind speed and direction.

Ammonia emission rates were calculated using a mass balance approach, the integrated horizontal flux (IHF) method^30^,^31^. The method is well-suited for small and well-defined experimental areas, and requires no corrections for atmospheric stability or the shape of the wind profile^32^. The emission rate, which is the vertical flux, was calculated by integrating the horizontal flux density across the vertical profile:


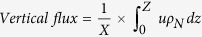


where *X* is the mean fetch (distance from edge of pen along the line of the mean wind direction to the centre mast) for the calculated period, *u* is the horizontal wind speed at height *z*, and *ρ*_*N*_ is the concentration of NH_3_ at height *z*. It is assumed that the horizontal flux is zero at the ground because the wind speed goes to zero there. The background concentrations at the height of 4 m are subtracted from the measured concentrations to get the *ρ*_*N*_ in the calculation. We reduced the calculated flux by 15%, based on empirical evidence from previous studies that the IHF method overestimates the true flux by 10–15%^33^,^34^.

Ammonia data was not available from 27^th^ November to 6^th^ December when the EC9842 analyser at the lignite pen malfunctioned. Following Junninen *et al.*^35^. We applied linear regression to compute cumulative NH_3_ fluxes for the period had missing data based on the data obtained 7 days prior to and 7 days after this period (Fig. 2). Similarly, there was some intermittent data lost (2^nd^, 3^rd^, 5^th^ and 6^th^ December) from the control pen. The diel pattern of NH_3_ emission was used to interpolate the daily fluxes of the four days that had missing hourly data points for the control pen.

According to the manufacturer^36^, the EC9842 analysers have a random error (precision) of 1% and a systematic error of 5% for measurements taken at a 5-minute interval. We calculated the total errors for the cumulative fluxes based on the nominal errors defined by the manufacturer using the approach of Moncrieff *et al.*^37^ (Fig. 2). Random errors had a minimal impact (accounting for approximately 1‰) on the cumulative flux^37^. In addition, we allowed a 20% systematic error in a sensitivity analysis as shown in Fig. 2, which still shows a significant difference between lignite and control treatments.

## Additional Information

**How to cite this article**: Chen, D. *et al.* A new cost-effective method to mitigate ammonia loss from intensive cattle feedlots: application of lignite. *Sci. Rep.* **5**, 16689; doi: 10.1038/srep16689 (2015).

## Figures and Tables

**Table 1:** Summary of predicted* and measured N content of feedlot manure.

		Control	Lignite
N intake	g head^−1^ d^−1^	358	358
Predicted N in excreta	g head^−1^ d^−1^	345−348	345−348
N retained in manure at the end of 40 days (±se)	kg head^−1^	5.3 ± 0.09	9.9 ± 0.14
Daily average NH_3_-N emission rate (±se); Including interpolated missing data	g head^−1^ d^−1^	156.4 ± 10.7	53.2 ± 6.4
Daily average NH_3_-N emission rate (±se); with measured data only	g head^−1^ d^−1^	149.7 ± 10.5	44.8 ± 6.5
Cumulative NH_3_-N emission over 40 days (5% systematic error)	kg head^−1^	6.26 ± 0.31	2.13 ± 0.11

^*^Based on: National Research Council. *Nutrient requirements of beef cattle.* National Academy Press Washington, DC, 1996.
